# Superparamagnetic iron oxide nanoparticle regulates microbiota–gut–inner ear axis for hearing protection

**DOI:** 10.1093/nsr/nwae100

**Published:** 2024-03-18

**Authors:** Zhanhang Guo, Yunhao Wu, Bo Chen, Mengdie Kong, Peng Xie, Yan Li, Dongfang Liu, Renjie Chai, Ning Gu

**Affiliations:** Jiangsu Key Laboratory for Biomaterials and Devices, School of Biological Science and Medical Engineering, Southeast University, Nanjing 210009, China; Medical Science and Technology Innovation Center, Shandong First Medical University & Shandong Academy of Medical Sciences, Jinan 250000, China; State Key Laboratory of Digital Medical Engineering, Department of Otolaryngology Head and Neck Surgery, Zhongda Hospital, School of Life Sciences and Technology, School of Medicine, Advanced Institute for Life and Health, Jiangsu Province High-Tech Key Laboratory for Bio-Medical Research, Southeast University, Nanjing 210096, China; Institute of Materials Science and Devices, School of Materials Science and Engineering, Suzhou University of Science and Technology, Suzhou 215009, China; State Key Laboratory of Digital Medical Engineering, Department of Otolaryngology Head and Neck Surgery, Zhongda Hospital, School of Life Sciences and Technology, School of Medicine, Advanced Institute for Life and Health, Jiangsu Province High-Tech Key Laboratory for Bio-Medical Research, Southeast University, Nanjing 210096, China; State Key Laboratory of Digital Medical Engineering, Department of Otolaryngology Head and Neck Surgery, Zhongda Hospital, School of Life Sciences and Technology, School of Medicine, Advanced Institute for Life and Health, Jiangsu Province High-Tech Key Laboratory for Bio-Medical Research, Southeast University, Nanjing 210096, China; Jiangsu Key Laboratory for Biomaterials and Devices, School of Biological Science and Medical Engineering, Southeast University, Nanjing 210009, China; Nurturing Center of Jiangsu Province for State Laboratory of AI Imaging & Interventional Radiology & Vascular Surgery, Department of Radiology, Medical School, Zhongda Hospital, Southeast University, Nanjing 210009, China; State Key Laboratory of Digital Medical Engineering, Department of Otolaryngology Head and Neck Surgery, Zhongda Hospital, School of Life Sciences and Technology, School of Medicine, Advanced Institute for Life and Health, Jiangsu Province High-Tech Key Laboratory for Bio-Medical Research, Southeast University, Nanjing 210096, China; Co-Innovation Center of Neuroregeneration, Nantong University, Nantong 226001, China; School of Medical Technology, Institute of Engineering Medicine, Beijing Institute of Technology, Beijing 100081, China; Department of Otolaryngology Head and Neck Surgery, Sichuan Provincial People's Hospital, University of Electronic Science and Technology of China, Chengdu 610072, China; Institute for Stem Cell and Regeneration, Chinese Academy of Sciences, Beijing 100101, China; Southeast university Shenzhen research institute, Shenzhen 518063, China; Jiangsu Key Laboratory for Biomaterials and Devices, School of Biological Science and Medical Engineering, Southeast University, Nanjing 210009, China; Cardiovascular Disease Research Center, Nanjing Drum Tower Hospital, Affiliated Hospital of Medical School, Medical School, Nanjing University, Nanjing 210093, China

**Keywords:** superparamagnetic iron oxide nanoparticle, hearing loss, gut microbiota, inflammation, sphingolipid metabolism, microbiota–gut–inner ear axis

## Abstract

Noise-induced hearing loss (NIHL) is a highly prevalent form of sensorineural hearing damage that has significant negative effects on individuals of all ages and there are no effective drugs approved by the US Food and Drug Administration. In this study, we unveil the potential of superparamagnetic iron oxide nanoparticle assembly (SPIOCA) to reshape the dysbiosis of gut microbiota for treating NIHL. This modulation inhibits intestinal inflammation and oxidative stress responses, protecting the integrity of the intestinal barrier. Consequently, it reduces the transportation of pathogens and inflammatory factors from the bloodstream to the cochlea. Additionally, gut microbiota-modulated SPIOCA-induced metabolic reprogramming in the gut–inner ear axis mainly depends on the regulation of the sphingolipid metabolic pathway, which further contributes to the restoration of hearing function. Our study confirms the role of the microbiota–gut–inner ear axis in NIHL and provides a novel alternative for the treatment of NIHL and other microbiota dysbiosis-related diseases.

## Teaser

This study presents a novel strategy to protect against noise-induced hearing loss by regulating the microbiota-gut-inner ear axis with iron oxide nanoparticles.

## INTRODUCTION

Hearing loss is one of the most common sensory deficiency diseases in the world. Noise-induced hearing loss (NIHL) is a progressive sensorineural hearing impairment caused by exposure to a harmful noise environment and has a profound impact on individuals of all ages [1]. Noise overexposure usually triggers cellular calcium dyshomeostasis, decreased mitochondrial membrane potential, inflammation and the accumulation of reactive oxygen species in cochlear hair cells, all of which result in an imbalance between oxidation and antioxidation, and lead to the death of hair cells [2,3]. Nowadays the treatment for NIHL mainly depends on hearing aids and cochlear implants, but the therapeutic effect is limited by the number and function of remaining cochlear hair cells and auditory nerves. Therefore, an urgent requirement has emerged to discover and innovate novel interventions for addressing NIHL.

The gut microbiota—a diverse microbial community located in the gastrointestinal tract—plays a pivotal role in several essential physiological processes that are critical to the overall health of the host. These processes include energy homeostasis, metabolism, gut epithelial health, immune activity, neurobehavioral development and interactions with other body organs [4–8]. It has been reported that the compositional variation in the intestinal flora is involved in the progression of hearing loss [9]. An inflammatory gut microenvironment induced by pathological stress may result in the disruption of the gut barrier and the translocation of pathogens, metabolites and pro-inflammatory factors derived from gut microbiota through the systemic circulation to other organs, including the inner ear [10]. Apart from the direct negative effects on cells in the inner ear, environmental noise stress has been reported to be related to gut microbiota dysregulation [11–13]. Therefore, the gut microbiota may be regarded as a potential target for the prevention and treatment of NIHL.

Currently, interventions for manipulating the gut microbiota are limited to the use of prebiotics and fecal microbiota transplantation (FMT) [14]. The former is hindered by variations in the ability of different bacteria to degrade prebiotics, making precise modulation of the gut microbial community challenging [15]. The latter carries potential risks of pathogen transmission during the FMT process [16]. It is of great significance to develop new, safe and effective strategies for the modulation of the gut microbiota.

**Figure 1.**
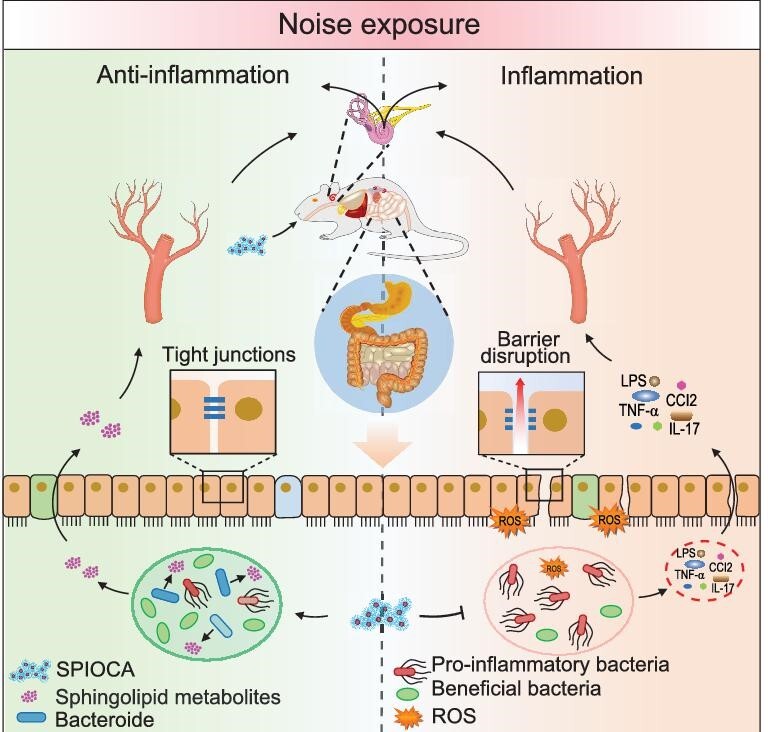
Schematic illustration of the mechanism through which SPIOCA regulates the microbiota–gut–inner ear axis for hearing protection.

In this work, we developed a pH-responsive superparamagnetic iron oxide nanoparticle assembly (SPIOCA) with the Food and Drug Administration (FDA)-approved excipient carboxymethyl cellulose (CMC) as the coating agent and investigated its regulatory effects on NIHL and intestinal dysbacteriosis. Based on its good biocompatibility and effectiveness in the modulation of the gut microbiota, we proposed that SPIOCA exhibits a protecting effect against NIHL through the modulation of the microbiota–gut–inner ear axis (Fig. 1). This study highlights a new strategy for preventing NIHL and the developed nano-iron oxide holds the potential as a therapeutic nanomedicine for the treatment of both NIHL and microbiota dysbiosis-related diseases.

## RESULTS AND DISCUSSION

### Preparation and characterization of SPIOCA

**Figure 2.**
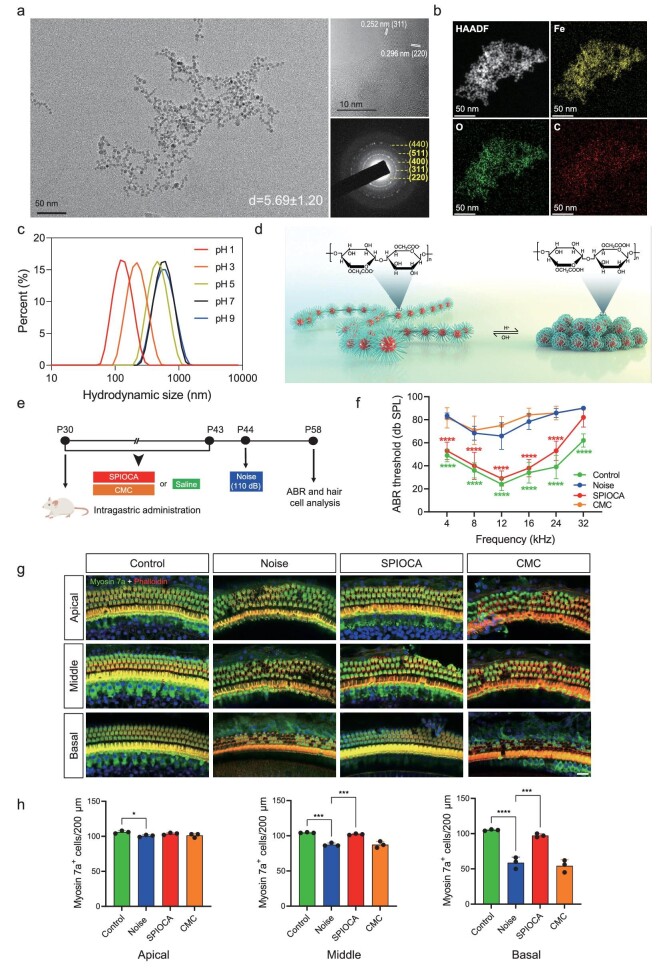
Protective effects of SPIOCA on NIHL. (a) TEM image, high-resolution TEM image and SAED pattern of SPIOCA. (b) HAADF–STEM image and the corresponding elemental maps showing the distribution of Fe, C and O. (c) The hydrodynamic diameter of SPIOCA was measured across a pH range of 1-9. (d) Schematic diagram of SPIOCA's pH responsiveness. (e) Schematic illustration of the animal experiments. (f) ABR thresholds were analysed to determine the effect of SPIOCA on NIHL (*n* = 6). (g) Hair cells in the apical, middle and basal turns in mouse cochleae were stained with myosin 7a, phalloidin and DAPI (4,6-diamidino-2-phenylindole). Scale bars: 20 μm. (h) Quantification of the number of cochlear hair cells per 200 μm in the apical, middle and basal turns (*n* = 3). *P < 0.05; **P < 0.01; ***P < 0.001; ****P < 0.0001.

The structural stability of nanomaterials in the gastrointestinal tract is crucial for preserving their functionality in regulating the gut microbiota [17]. For orally delivered iron oxide nanomaterials, maintaining their structural stability in the highly acidic gastric environment is of utmost importance. To address this requirement, the FDA-approved excipient CMC—a water-soluble derivative of cellulose—was employed as a coating agent to prepare SPIOCA for oral administration using a simple one-pot co-precipitation method. Particle size control was achieved by varying the ratios of CMC/ferrous chloride/ferric chloride (Fig. S1a–c) and the product obtained with a ratio of 4:2:1 exhibited the most suitable crystallinity and stability (Fig. S1d). The morphology of the SPIOCA was characterized by using a transmission electron microscope (TEM). Due to the strong interaction between CMC coatings, the iron oxide nanoparticles in SPIOCA exist in the form of small assemblies. As shown in Fig. 2a, the resulting iron oxide nanoparticles exhibited regular spherical shapes with a diameter of ∼5.69 nm, displaying distinct lattice fringes with lattice spacings of 0.252 and 0.296 nm, corresponding to the (311) and (220) crystal planes of γ-Fe_2_O_3_, respectively. Selected area electron diffraction (SAED) revealed diffraction rings of SPIOCA corresponding to the crystal planes of γ-Fe_2_O_3_. X-ray diffraction analysis showed absorption peaks at 30.3°, 35.6°, 43.5°, 53.3°, 57.3° and 63.1°, corresponding to the (220), (311), (400), (422), (511) and (440) crystal planes of γ-Fe_2_O_3_, indicating the presence of a magnetic spinel phase (Fig. S2a) (JCPDS: 39–1346). Fourier transform infrared (FTIR) spectroscopy of SPIOCA exhibited strong peaks at 2876, 1573 and 1024 cm^−1^, corresponding to the stretching and bending vibrations of C–H, –C=O and C–O–C bonds in CMC (Fig. S2b). Additionally, a stretching vibration peak at 585 cm^−1^ corresponding to the Fe–O bond was observed. In addition, iron, oxygen and carbon elements are evenly distributed in SPIOCA (Fig. 2b). These results demonstrate the successful coordination between iron oxide and CMC. Thermogravimetric analysis (TGA) showed a mass loss of ∼45% for SPIOCA in the temperature range of 150–570°C (Fig. S2c) and this could be attributed to the thermal decomposition of the CMC coating. SPIOCA exhibits excellent superparamagnetic behavior overall (Fig. S3a) and the surface is negatively charged due to the coating of CMC (Fig. S3b). The hydrodynamic diameter of SPIOCA was 430 nm with a polydispersity index (PDI) of 0.199 and there were no significant changes in size and PDI after 14 days (Fig. S4a), demonstrating the excellent colloidal stability of SPIOCA in an aqueous medium.

The pH-responsive property of SPIOCA was evaluated in the range of pH 1–9. As shown in Fig. 2c, the hydrodynamic diameter of SPIOCA gradually decreases with the decrease in pH within the pH range of 1–7. The conformation of SPIOCA contracted in the acidic solution due to the protonation of CMC (Fig. 2d). The pH-responsive behavior reduces the exposure of the iron oxide core and protects SPIOCA from degradation in the highly acidic gastric environment. After 2 hours of incubation in simulated gastric fluid, SPIOCA released only 5.5 μg/mL of iron ions, with approximately half of the release observed from bare γ-Fe_2_O_3_ nanoparticles (Fig. S4b). This indicates that SPIOCA exhibits superior stability.

### Biocompatibility analysis of SPIOCA *in vitro* and *in vivo*

Histological analysis was performed to investigate the pathological features in the main organs harvested from C57BL/6 mice after 2 weeks of oral administration of SPIOCA. Based on the H&E (Hematoxylin and Eosin) staining results, there were no significant lesions observed in the heart, liver, spleen, lungs, kidneys, stomach or intestinal tissues of the SPIOCA treatment group compared with the control group (Fig. S5). To determine the effect of SPIOCA on the function of the liver and kidney, the levels of alanine aminotransferase, aspartate aminotransferase, alkaline phosphatase, urea and creatinine were measured. Compared with the control group, the values of these indicators remained in the normal range after oral administration of the nanoparticles (Fig. S6a–e). The cytotoxicity of SPIOCA against Caco-2 cells was evaluated with a series of doses from 10 to 200 μg of Fe/mL via a Cell Counting Kit-8 cell (CCK-8) assay. As shown in Fig. S6f, no significant cytotoxicity was observed even at an iron concentration of ≤200 μg/mL. The above results suggest that SPIOCA exhibits good biocompatibility at both the cellular and organismal levels, and thus is a safe oral agent.

### Protection against NIHL

We next investigate the therapeutic efficacy of SPIOCA against NIHL in mice. Postnatal day (P)30 C57 mice received an intragastric administration of SPIOCA (26 mg/kg per day of iron dose) and CMC, while the control group was given the same volume of saline. Two weeks later, mice were subjected to white noise (110 dB) exposure for 2 hours, and auditory brainstem response (ABR) threshold measurements and immunofluorescence experiments in cochlear hair cells were performed 2 weeks later (Fig. 2e). ABR analysis showed that oral administration of SPIOCA had a protective effect against NIHL (Fig. 2f). The cochlear outer hair cells (OHCs) are mechanosensory cells that selectively amplify auditory inputs, whereas inner hair cells are responsible for transmitting acoustic information to the brain through ribbon synapses [18,19]. When the ear receives sound signals, different sound frequencies are separated along the cochlea, with each hair cell being tuned to a narrow frequency range [20]. Dysfunction or loss of OHCs is regarded as a predominant cause of hearing deficiency. In the present study, mouse cochlear hair cells were stained with myosin 7a and phalloidin, and, after noise exposure, we observed an obvious loss of OHCs in the apical, middle and basal turns, while pretreatment with SPIOCA could promote OHC survival (Fig. 2g and h). Taken together, these results suggest that SPIOCA has effective therapeutic effects against NIHL.

### Amelioration of cochlear inflammation

**Figure 3.**
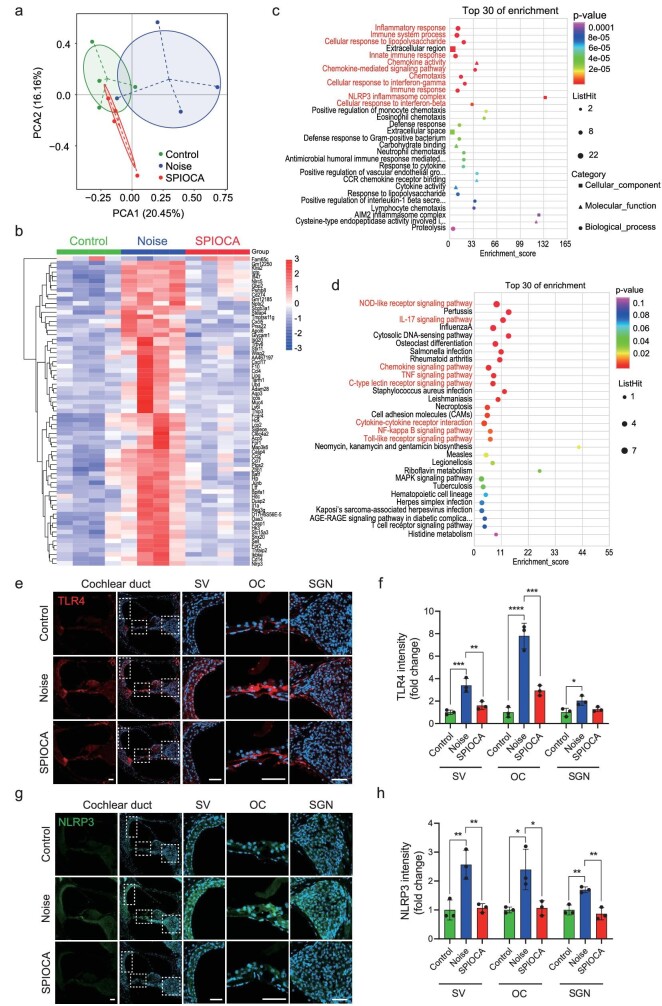
Cochlear inflammation of mice in different groups. (a) PCA plot showing the clustering of cochleae in different groups. (b) Heat map of cochlear gene expression obtained by mRNA sequencing comparing four independent samples in different groups. (c) GO enrichment of the top 30 differentially expressed genes. (d) KEGG enrichment of the top 30 differentially expressed genes. (e) Immunofluorescence of TLR4 in cochlear duct. Scale bars: 50 μm. (f) Quantification of TLR4 expression in cochlear stria vascularis (SV), organ of Corti (OC), spiral ganglion neuron (SGN). (g) Immunofluorescence of NLRP3 in cochlear duct. Scale bars: 50 μm. (h) Quantification of NLRP3 expression in Cochlear SV, OC, SGN. *P < 0.05; **P < 0.01; ***P < 0.001; ****P < 0.0001.

To delineate the protective mechanisms of SPIOCA, we performed genome-wide transcriptional profiling of the mouse cochlea by using RNA-sequencing (RNA-seq). Principle component analysis (PCA) showed that the noise-exposed group displayed a shift clustering that was distinct from the control group, while the SPIOCA-treated group was more closely clustered to the control group (Fig. 3a). RNA-seq identified 69 significantly altered genes, 68 of which were upregulated and 1 was downregulated in the cochlea of the noise-exposed group versus the control group, and the altered expression of all 69 genes was reversed by SPIOCA treatment (Fig. 3b). Gene Ontology (GO) analysis and Kyoto Encyclopedia of Genes and Genomes (KEGG) pathway analysis were conducted to determine the functions and pathways involved in SPIOCA regulation. Among the top 30 enrichments, we found that immuno-inflammatory responses and related pathways such as the NOD-like receptor signaling pathway, interleukin (IL)-17 signaling pathway, tumor necrosis factor (TNF) signaling pathway, NF-κB signaling pathway and Toll-like receptor signaling pathway were regulated by SPIOCA treatment (Fig. 3c and d). TLR4 is an important receptor for recognizing lipopolysaccharide (LPS) and activation of TLR4 contributes to inflammatory signaling [21]. Its activation could upregulate the production of pro-inflammatory cytokines in mouse cochlea [22]. NLRP3 is an innate cytosolic immune signaling receptor and its activation leads to caspase 1-mediated proteolytic activation of the IL-1β family of cytokines, thus resulting in an inflammatory, pyroptotic cell death [23]. It has been reported that NLRP3 inhibition alleviates inflammatory phenotypes in the inner ear and protects against inflammation-mediated sensorineural hearing loss [24]. Here we found that SPIOCA could reverse noise-induced activation of TLR4 and NLRP3 in mouse cochlea (Fig. 3e–h and Fig. S7), which demonstrated that SPIOCA protects against NIHL mainly by the inhibition of cochlear immuno-inflammatory responses.

### Modulation of the gut microbiome

**Figure 4.**
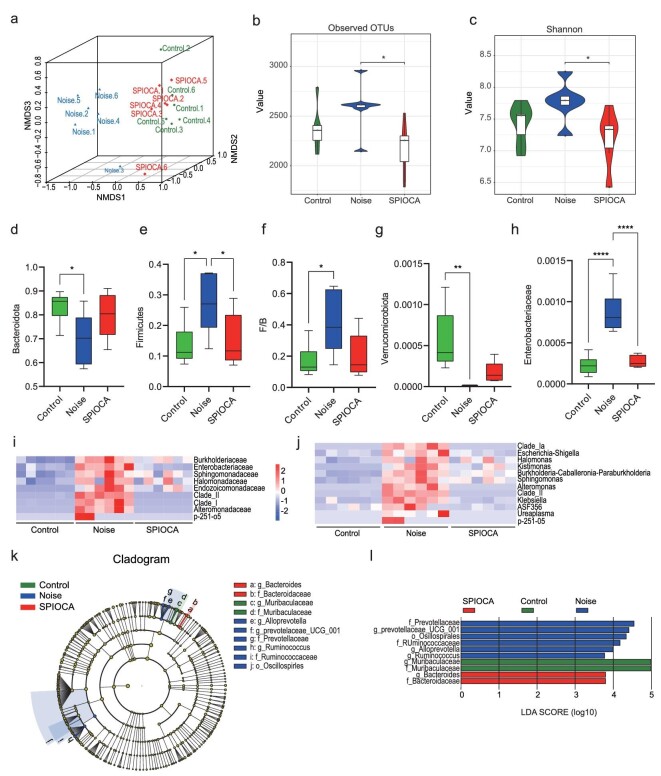
SPIOCA modulates the composition of the gut microbiota. (a) NMDS plot illustrating the gut microbiome β-diversity. (b and c) Estimation of the microbial community observed (b) OTU richness and (c) α-diversity (Shannon index). (d–g) Relative abundance of *Bacteroidota, Firmicutes, Verrucomicrobiota* and the F/B ratio at the phylum-level taxonomy after different treatments. (h) Relative abundance of *Enterobacteriaceae* at the family-level taxonomy after different treatments. Heat map of the relative abundance at the (i) family-level and (j) genus-level taxa (rows) for each mouse (columns). (k) Cladogram based on LEfSe analysis showing the community composition of the gut microbiota in mice with different treatments. (l) Discriminative taxa determined by LEfSe among different groups (log_10_ LDA > 3.5). *P < 0.05; **P < 0.01; ****P < 0.0001.

SPIOCA is mainly distributed in the gastrointestinal tract after oral administration (Fig. S8). The internal inflammatory responses in various diseases have been reported to be associated with alterations in the gut microbiota [25–28]. Here, we investigated whether the suppressive effect on cochlear inflammation is linked to the regulation of the gut microbiota. The gut microbiota composition after intragastric SPIOCA administration was analysed using 16S rRNA gene amplicon sequencing in the colonic contents. Non-metric multidimensional scaling (NMDS) analysis was used to visualize the differences in the fecal microbiota structure among different groups. Plots representing the noise group were distributed in the left-hand space, while the control group and SPIOCA group were located in the right-hand space, indicating that noise exposure significantly altered the gut microbiota composition. However, SPIOCA administration mitigated the drastic shift in gut microbiota (Fig. 4a). SPIOCA treatment also significantly inhibited the noise-induced increase in bacterial richness (observed operational taxonomic unit (OTU) richness) and diversity (Shannon index) (Fig. 4b and c). *Firmicutes* and *Bacteroidetes* are the dominant microflora in gut microbiota and the *Firmicutes*/*Bacteroidetes* (F/B) ratio has been associated with several pathological conditions [29]. We observed that the F/B ratio was significantly higher in mice in the noise-exposed group compared with the control group, while it was decreased in the SPIOCA-treated group (Fig. 4d–f). *Verrucomicrobiota* is involved in maintaining intestinal integrity [30] and we found that noise exposure remarkably reduced the abundance of *Verrucomicrobiota*, while there was an improvement in the SPIOCA group, indicating that SPIOCA might have a protective effect on gut barrier integrity (Fig. 4g). It has been reported that the expansion of facultative anaerobic *Enterobacteriaceae* is a common marker of microbiota dysbiosis and the bacteria-derived endotoxin may result in systemic inflammation [31,32]. In the current study, we found that SPIOCA significantly suppressed the increased abundance of *Enterobacteriaceae* in NIHL mice (Fig. 4h). We next analysed the significantly changed gut microbiota composition at the family and genus levels, and the significant microbial changes between different groups are shown in Fig. 4i and j. We observed that SPIOCA treatment could remarkably reduce noise-induced increases in the abundance of maleficent bacteria such as *Escherichia-Shigella, Kiebsiella* and *Ureaplasma*. The increased relative abundance of these bacteria has been proven to be closely associated with upregulated intestinal inflammation, intestinal barrier disruption and internal infections [33–35]. Note that, unlike excessive exposure to iron ions leading to dysbiosis of the gut microbiota [36], SPIOCA demonstrates a positive modulatory effect on the gut microbiota. The linear discriminant analysis effect size (LEfSe) is used to assess the alterations of microbiota composition in each group and the enriched taxa in the mouse microbiota are represented in a cladogram (Fig. 4k). The greatest difference from the phylum to the genus level in microbiota was identified using a linear discriminant analysis (LDA) score, which showed that the relative abundance of *Bacteroides* was enriched in the SPIOCA group (Fig. 4l). Collectively, these results suggested that the benefits of SPIOCA can be attributed to the modulation of the gut microbiome.

### Inhibition of intestinal inflammation and restoration of the gut and the blood–labyrinth barrier

**Figure 5.**
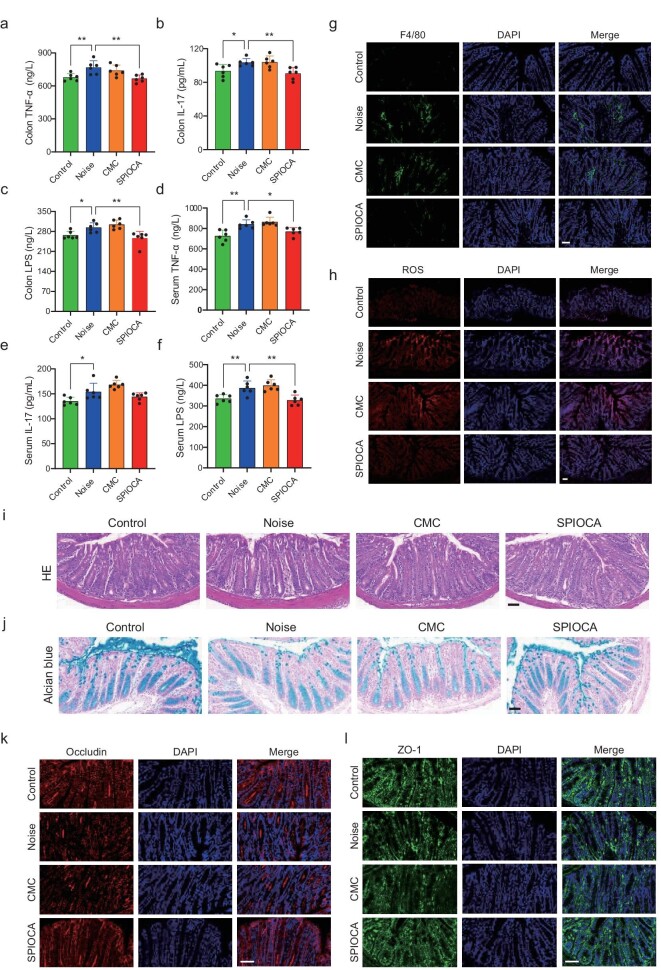
SPIOCA inhibits colonic inflammation and restores gut barrier integrity. (a–c) Detection of TNF-α, IL-17 and LPS in colon tissue by using ELISA (Enzyme-Linked Immunosorbent Assay) kits. (d–f) Detection of TNF-α, IL-17 and LPS in serum by using ELISA kits. (g) Immunostaining of F4/80 in colon tissue. (h) Immunostaining images of ROS levels in colon tissue. (i) HE staining of colon tissue with different treatments. (j) Alcian blue staining of colon tissue with different treatments. Immunostaining of (k) occludin and (l) ZO-1 in colon tissue in different groups. Scale bars: 50 μm. *P < 0.05; **P < 0.01.

The microbiota–gut–brain axis plays a crucial role in the bidirectional association between the gut and the brain, contributing to the pathogenesis of various neurological disorders [37]. Gut microbial dysbiosis-induced increase in pro-inflammatory cytokines that are produced by the pro-inflammatory microbiome leads to the increased permeability of the intestinal barrier, and then the circulating pro-inflammatory factors disrupt the blood–brain barrier (BBB) integrity and result in neuroinflammation [38]. The blood–labyrinth barrier (BLB) in the inner ear, which resembles the BBB, protects the cochlea from invasion by pathogens, toxins and mediators of inflammation [39]. The properties of the BLB make the inner ear particularly susceptible to influences from gut microbiota. Here we observed that noise exposure resulted in the elevation of inflammatory factors such as TNF-α, IL-17 and LPS in both colon tissues and serum, which was attenuated by SPIOCA treatment (Fig. 5a–f). Furthermore, by immunostaining F4/80 and reactive oxygen species (ROS) in colon tissue, we found that SPIOCA could effectively reduce the inflammation and oxidative stress induced by noise exposure (Fig. 5g and h, and Fig. S9a and b). HE staining showed that there was no significant change in different groups (Fig. 5i), indicating that the noise stress was not severe enough to induce pathological lesions in colon tissue. However, we observed a thinner mucus layer and reduced expression of tight junction proteins such as occludin and ZO-1 after noise exposure in the colon section, and these were alleviated by SPIOCA treatment (Fig. 5j–l and Fig. S9c and d), suggesting that SPIOCA exerted a protective effect on noise-induced gut barrier disruption. It is reported that inflammatory factors can cause significant disruption of BLB [40] and the increased inflammatory factors in the circulation may damage the cochlea through the broken BLB. We further found that noise exposure disrupted the integrity of BLB through the dextran leakage experiment, while there was an improvement after SPIOCA treatment (Fig. S10). Taken together, our results suggest that SPIOCA protects against noise-induced cochlear inflammation by inhibiting the pro-inflammatory factors via the microbiota–gut–inner ear axis.

### Regulation of sphingolipid metabolism in gut–inner ear axis

**Figure 6.**
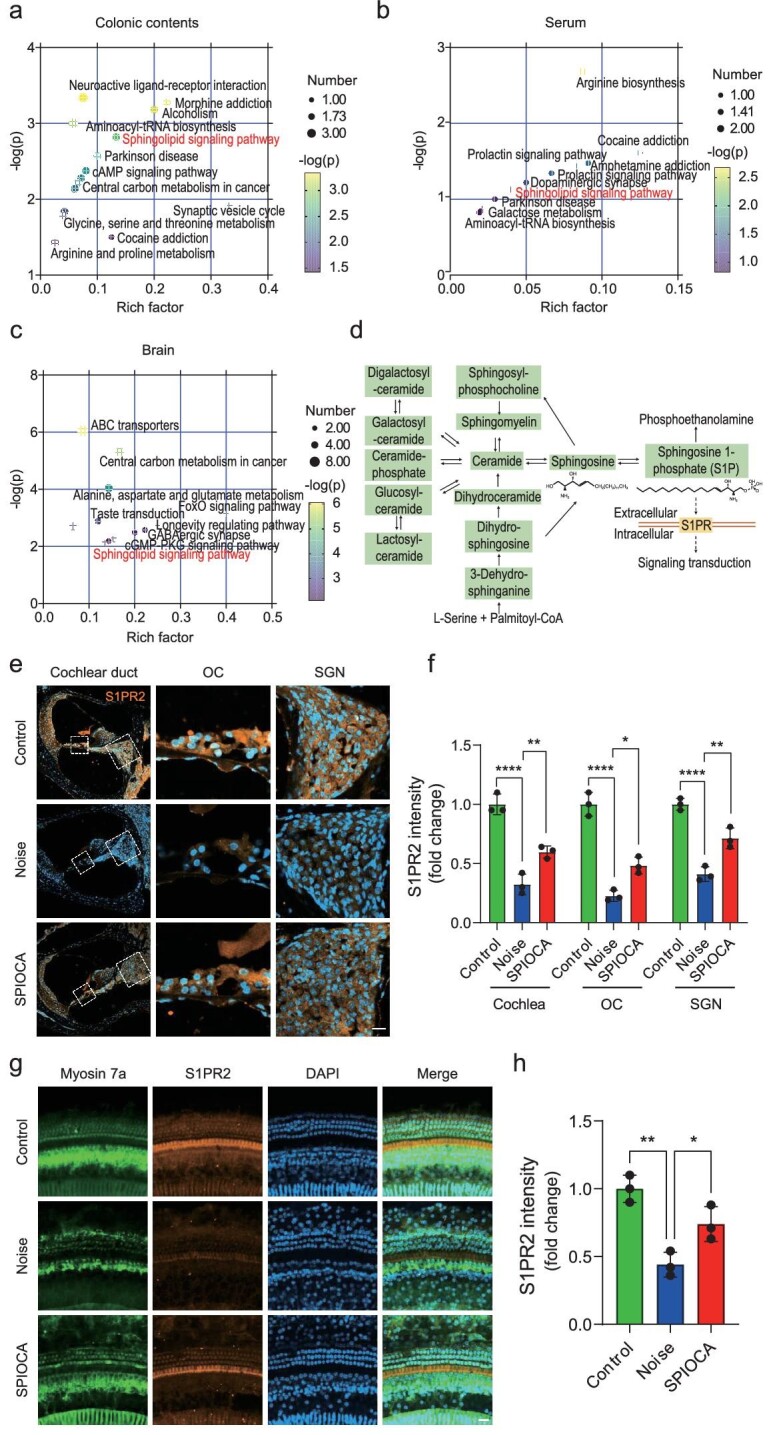
SPIOCA regulates microbiota-derived sphingolipid metabolism. (a–c) Pathway enrichment analysis of the significantly changed metabolites in colonic contents, serum and brain tissues. (d) Graphical representation of sphingolipid metabolism. (e) Immunofluorescence of the expression of S1PR2 in the mouse cochlea (*n* = 3). Scale bars: 20 μm. (f) Quantification of S1PR2 expression in the cochlea. OC, organ of Corti; SGN, spiral ganglion neuron. (g) Immunofluorescence of S1PR2 expression in cochlear hair cells. Hair cells were stained for myosin 7a (*n* = 3). Scale bars: 20 μm. (h) Quantification of S1PR2 expression in the hair cells. *P < 0.05; **P < 0.01; ****P < 0.0001.

Microbial metabolites may influence neurodegenerative disorders by modulating many signaling pathways [41–43]. In the present study, untargeted metabolomics analysis was performed to determine the metabolic alterations in response to SPIOCA administration in colonic contents, serum and brain samples by using gas chromatography and mass spectrometry. Orthogonal partial least squares-discriminant analysis showed a significant separation of clusters between different groups (Control vs. Noise and Noise vs. SPIOCA) (Fig. S11a–f). Heat-map analysis showed a significant change in metabolites among three groups, with a total of 23 metabolites in mouse colonic contents, 23 metabolites in serum and 56 metabolites in brain tissues (Fig. S11g–i). The metabolomic map displays the significantly enriched pathways that were regulated by SPIOCA administration in mouse colonic contents, serum and brain tissues (Fig. 6a–c). Interestingly, the sphingolipid signaling pathway was prominently influenced by SPIOCA in all three samples (Fig. 6a–c). Sphingolipids are both structural lipids embedded in eukaryotic membrane bilayers and a fundamental class of signaling molecules mediating multiple biological functions [44]. Sphingolipids are readily synthesized by gut microbes of the genera *Bacteroides* [45] and our results presented in Fig. 4l also showed that *Bacteroides* was significantly enriched in the SPIOCA group, indicating a moderating effect of SPIOCA on sphingolipid metabolism. Among all sphingolipids, S1P is the most well-characterized intercellular signaling molecule and it plays a pivotal role in regulating various physiological functions through S1P receptors (S1PRs) (Fig. 6d). It has been reported that, among the five specific G protein-coupled S1PRs (S1PR1–S1PR5), only S1PR1–S1PR3 is expressed in the cochlea and that S1PR2 inhibition promotes the ototoxicity of gentamicin, thus highlighting the important role of S1PR2 in auditory function [46]. Here, we observed that noise exposure suppressed the expression of S1PR2 in both cochlear hair cells and spiral ganglion neurons, which was reversed by SPIOCA treatment (Fig. 6e–h). Meanwhile, numerous studies have demonstrated that S1P–S1PR signaling is involved in mediating immune and inflammatory processes [47–50]. In line with this, our results also showed that SPIOCA could suppress the immune and inflammatory responses in the cochlea triggered by noise, indicating that SPIOCA-mediated sphingolipid metabolism in the gut–inner ear axis plays a significant role in preventing NIHL.

## CONCLUSION

In summary, we developed an orally administered iron oxide nanomaterial (SPIOCA) with pH responsiveness and excellent biocompatibility, and evaluated its protective effects against noise-induced gut microbiota dysbiosis and hearing loss. Oral administration of SPIOCA can inhibit the harmful bacteria proliferation induced by noise, thereby protecting against intestinal barrier damage caused by gut inflammation and oxidative stress, and avoiding systemic inflammatory responses. This is closely related to the inhibition of cochlear inflammation induced by noise, thus preserving hearing function. Additionally, SPIOCA specifically induced an increase in the abundance of sphingolipid-producing *Bacteroides*, thus regulating the sphingolipid metabolism in the gut–inner ear axis and protecting hearing.

This study demonstrates a novel mechanism whereby the gut microbiota influences cochlear inflammation, providing valuable insights into the field of hearing protection. Additionally, it reveals, for the first time, the positive restorative effect of iron oxide nanomaterials on dysbiotic gut microbiota. By targeting the modulation of gut microbiota, SPIOCA has the potential to become an effective nanomedicine for the treatment of NIHL. Nevertheless, further in-depth research is still required to elucidate the mechanisms underlying the impact of SPIOCA on gut microbiota changes. The SPIOCA developed in this work is simple to prepare, and all components are FDA-approved for clinical use, making it a promising treatment for other diseases associated with gut microbiota dysbiosis.

## Supplementary Material

nwae100_Supplemental_File

## References

[1] Basner M, Babisch W, Davis A et al. Auditory and non-auditory effects of noise on health. Lancet 2014; 383: 1325–32. 24183105 10.1016/S0140-6736(13)61613-X PMC3988259

[2] Delmaghani S, Defourny J, Aghaie A et al. Hypervulnerability to sound exposure through impaired adaptive proliferation of peroxisomes. Cell 2015; 163: 894–906. 10.1016/j.cell.2015.10.023 26544938

[3] Wu F, Xiong H, Sha SH. Noise-induced loss of sensory hair cells is mediated by ROS/AMPKα pathway. Redox Biol 2020; 29: 101406. 10.1016/j.redox.2019.101406 31926629 PMC6933152

[4] Wang ZN, Zhao YZ. Gut microbiota derived metabolites in cardiovascular health and disease. Protein Cell 2018; 9: 416–31. 10.1007/s13238-018-0549-0 29725935 PMC5960473

[5] Wu ZH, Huang SM, Li TT et al. Gut microbiota from green tea polyphenol-dosed mice improves intestinal epithelial homeostasis and ameliorates experimental colitis. Microbiome 2021; 9: 184. 10.1186/s40168-021-01115-9 34493333 PMC8424887

[6] Wastyk HC, Fragiadakis GK, Perelman D et al. Gut-microbiota-targeted diets modulate human immune status. Cell 2021; 184: 4137–53. 10.1016/j.cell.2021.06.019 34256014 PMC9020749

[7] Sharon G, Cruz NJ, Kang DW et al. Human gut microbiota from autism spectrum disorder promote behavioral symptoms in mice. Cell 2019; 177: 1600–18. 10.1016/j.cell.2019.05.004 31150625 PMC6993574

[8] Cryan JF, O'Riordan KJ, Cowan CSM et al. The microbiota-gut-brain axis. Physiol Rev 2019; 99: 1877–2013. 10.1152/physrev.00018.2018 31460832

[9] Kondo T, Saigo S, Ugawa S et al. Prebiotic effect of fructo-oligosaccharides on the inner ear of DBA/2 J mice with early-onset progressive hearing loss. J Nutr Biochem 2020; 75: 108247. 10.1016/j.jnutbio.2019.108247 31707282

[10] Kociszewska D, Chan JF, Thorne PR et al. The link between gut dysbiosis caused by a high-fat diet and hearing loss. Int J Mol Sci 2021; 22: 13177. 10.3390/ijms222413177 34947974 PMC8708400

[11] Cui B, Su DH, Li WL et al. Effects of chronic noise exposure on the microbiome-gut-brain axis in senescence-accelerated prone mice: implications for Alzheimer's disease. J Neuroinflammation 2018; 15: 190. 10.1186/s12974-018-1223-4 29933742 PMC6015475

[12] Chi HM, Cao W, Zhang M et al. Environmental noise stress disturbs commensal microbiota homeostasis and induces oxi-inflammation and AD-like neuropathology through epithelial barrier disruption in the EOAD mouse model. J Neuroinflammation 2021; 18: 9. 10.1186/s12974-020-02053-3 33407614 PMC7789697

[13] Karl JP, Hatch AM, Arcidiacono SM et al. Effects of psychological, environmental and physical stressors on the gut microbiota. Front Microbiol 2018; 9: 2013. 10.3389/fmicb.2018.02013 30258412 PMC6143810

[14] Fassarella M, Blaak EE, Penders J et al. Gut microbiome stability and resilience: elucidating the response to perturbations in order to modulate gut health. Gut 2021; 70: 595–605. 10.1136/gutjnl-2020-321747 33051190

[15] Mills S, Stanton C, Lane JA et al. Precision nutrition and the microbiome. Part I: Current state of the science. Nutrients 2019; 11: 923. 10.3390/nu11040923 31022973 PMC6520976

[16] Rahman RA, Lamarca A, Hubner RA et al. The microbiome as a potential target for therapeutic manipulation in pancreatic cancer. Cancers 2021; 13: 3779. 10.3390/cancers13153779 34359684 PMC8345056

[17] Lee Y, Kamada N, Moon JJ. Oral nanomedicine for modulating immunity, intestinal barrier functions, and gut microbiome. Adv Drug Deliv Rev 2021; 179: 114021. 10.1016/j.addr.2021.114021 34710529 PMC8665886

[18] Ashmore J . Outer hair cells and electromotility. Csh Perspect Med 2019; 9: a033522. 10.1101/cshperspect.a033522 PMC6601450 30181355

[19] Fettiplace R . Hair cell transduction, tuning, and synaptic transmission in the mammalian cochlea. Compr Physiol 2017; 7: 1197–227. 10.1002/cphy.c160049 28915323 PMC5658794

[20] Liu S, Wang SF, Zou LZ et al. Mechanisms in cochlear hair cell mechano-electrical transduction for acquisition of sound frequency and intensity. Cell Mol Life Sci 2021; 78: 5083–94. 10.1007/s00018-021-03840-8 33871677 PMC11072359

[21] Ryu JK, Kim SJ, Rah SH et al. Reconstruction of LPS transfer cascade reveals structural determinants within LBP, CD14, and TLR4-MD2 for efficient LPS recognition and transfer. Immunity 2017; 46: 38–50. 10.1016/j.immuni.2016.11.007 27986454

[22] Oh GS, Kim HJ, Choi JH et al. Activation of lipopolysaccharide-TLR4 signaling accelerates the ototoxic potential of cisplatin in mice. J Immunol 2011; 186: 1140–50. 10.4049/jimmunol.1002183 21148032

[23] Mangan MSJ, Olhava EJ, Roush WR et al. Targeting the NLRP3 inflammasome in inflammatory diseases. Nat Rev Drug Discov 2018; 17: 588–606. 10.1038/nrd.2018.97 30026524

[24] Ma JH, Lee E, Yoon SH et al. Therapeutic effect of NLRP3 inhibition on hearing loss induced by systemic inflammation in a CAPS-associated mouse model. Ebiomedicine 2022; 82: 104184. 10.1016/j.ebiom.2022.104184 35870427 PMC9307460

[25] Pickard JM, Zeng MY, Caruso R et al. Gut microbiota: role in pathogen colonization, immune responses, and inflammatory disease. Immunol Rev 2017; 279: 70–89. 10.1111/imr.12567 28856738 PMC5657496

[26] Jamar G, Ribeiro DA, Pisani LP. High-fat or high-sugar diets as trigger inflammation in the microbiota-gut-brain axis. Crit Rev Food Sci Nutr 2021; 61: 836–54. 10.1080/10408398.2020.1747046 32267169

[27] Schachter J, Martel J, Lin CS et al. Effects of obesity on depression: a role for inflammation and the gut microbiota. Brain Behav Immun 2018; 69: 1–8. 10.1016/j.bbi.2017.08.026 28888668

[28] Chen X, Wu R, Li L et al. Pregnancy-induced changes to the gut microbiota drive macrophage pyroptosis and exacerbate septic inflammation. Immunity 2023; 56: 336–52. 10.1016/j.immuni.2023.01.015 36792573

[29] Jandhyala SM, Talukdar R, Subramanyam C et al. Role of the normal gut microbiota. World J Gastroenterol 2015; 21: 8787–803. 10.3748/wjg.v21.i29.8787 26269668 PMC4528021

[30] Geerlings SY, Kostopoulos I, de Vos WM et al. Akkermansia muciniphila in the human gastrointestinal tract: when, where, and how? Microorganisms 2018; 6: 75. 10.3390/microorganisms6030075 30041463 PMC6163243

[31] Wang H, Zhang M, Li J et al. Gut microbiota is causally associated with poststroke cognitive impairment through lipopolysaccharide and butyrate. J Neuroinflammation 2022; 19: 76. 10.1186/s12974-022-02435-9 35379265 PMC8981610

[32] Shin NR, Whon TW, Bae JW. Proteobacteria: microbial signature of dysbiosis in gut microbiota. Trends Biotechnol 2015; 33: 496–503. 10.1016/j.tibtech.2015.06.011 26210164

[33] Lee KS, Jeong YJ, Lee MS. Shiga toxins and gut microbiota interactions. Toxins 2021; 13: 416. 10.3390/toxins13060416 34208170 PMC8230793

[34] Nakamoto N, Sasaki N, Aoki R et al. Gut pathobionts underlie intestinal barrier dysfunction and liver T helper 17 cell immune response in primary sclerosing cholangitis. Nat Microbiol 2019; 4: 492–503. 10.1038/s41564-018-0333-1 30643240

[35] Wolfs TGA, Kallapur SG, Knox CL et al. Antenatal ureaplasma infection impairs development of the fetal ovine gut in an IL-1-dependent manner. Mucosal Immunol 2013; 6: 547–56. 10.1038/mi.2012.97 23149664

[36] Kortman GAM, Raffatellu M, Swinkels DW et al. Nutritional iron turned inside out: intestinal stress from a gut microbial perspective. FEMS Microbiol Rev 2014; 38: 1202–34. 10.1111/1574-6976.12086 25205464

[37] Socala K, Doboszewska U, Szopa A et al. The role of microbiota-gut-brain axis in neuropsychiatric and neurological disorders. Pharmacol Res 2021; 172: 105840. 10.1016/j.phrs.2021.105840 34450312

[38] Agirman G, Yu KB, Hsiao EY. Signaling inflammation across the gut-brain axis. Science 2021; 374: 1087–92. 10.1126/science.abi6087 34822299

[39] Hirose K, Hartsock JJ, Johnson S et al. Systemic lipopolysaccharide compromises the blood-labyrinth barrier and increases entry of serum fluorescein into the perilymph. J Assoc Res Otolaryngol 2014; 15: 707–19. 10.1007/s10162-014-0476-6 24952083 PMC4164684

[40] Sekulic M, Puche R, Bodmer D et al. Human blood-labyrinth barrier model to study the effects of cytokines and inflammation. Front Mol Neurosci 2023; 16: 1243370. 10.3389/fnmol.2023.1243370 37808472 PMC10551159

[41] Dalile B, Van Oudenhove L, Vervliet B et al. The role of short-chain fatty acids in microbiota-gut-brain communication. Nat Rev Gastroenterol Hepatol 2019; 16: 461–78. 10.1038/s41575-019-0157-3 31123355

[42] Jenkins TA, Nguyen JCD, Polglaze KE et al. Influence of tryptophan and serotonin on mood and cognition with a possible role of the gut-brain axis. Nutrients 2016; 8: 56. 10.3390/nu8010056 26805875 PMC4728667

[43] Mertens KL, Kalsbeek A, Soeters MR et al. Bile acid signaling pathways from the enterohepatic circulation to the central nervous system. Front Neurosci 2017; 11: 617. 10.3389/fnins.2017.00617 29163019 PMC5681992

[44] Hannun YA, Obeid LM. Sphingolipids and their metabolism in physiology and disease. Nat Rev Mol Cell Biol 2018; 19: 175–91. 10.1038/nrm.2017.107 29165427 PMC5902181

[45] Brown EM, Ke XB, Hitchcock D et al. Bacteroides-derived sphingolipids are critical for maintaining intestinal homeostasis and symbiosis. Cell Host Microbe 2019; 25: 668–80. 10.1016/j.chom.2019.04.002 31071294 PMC6544385

[46] Nakayama M, Tabuchi K, Hoshino T et al. The influence of sphingosine-1-phosphate receptor antagonists on gentamicin-induced hair cell loss of the rat cochlea. Neurosci Lett 2014; 561: 91–5. 10.1016/j.neulet.2013.12.063 24397911

[47] Baeyens A, Bracero S, Chaluvadi VS et al. Monocyte-derived S1P in the lymph node regulates immune responses. Nature 2021; 592: 290–5. 10.1038/s41586-021-03227-6 33658712 PMC8475585

[48] Pérez-Jeldres T, Alvarez-Lobos M, Rivera-Nieves J. Targeting sphingosine-1-phosphate signaling in immune-mediated diseases: beyond multiple sclerosis. Drugs 2021; 81: 985–1002. 10.1007/s40265-021-01528-8 33983615 PMC8116828

[49] Nagahashi M, Yamada A, Katsuta E et al. Targeting the SphK1/S1P/S1PR1 axis that links obesity, chronic inflammation, and breast cancer metastasis. Cancer Res 2018; 78: 1713–25. 10.1158/0008-5472.CAN-17-1423 29351902 PMC6945803

[50] Wang M . Targeting perivascular S1P attenuates inflammation. Nat Rev Nephrol 2022; 18: 679. 10.1038/s41581-022-00637-1 36131004

