# Supplementary material for: Superparamagnetic iron oxide nanoparticle regulates microbiota–gut–inner ear axis for hearing protection
Source: Natl Sci Rev. 2024 Mar 18;11(6):nwae100. doi: 10.1093/nsr/nwae100 (PMC11067960; doi:10.1093/nsr/nwae100)
Supplement: nwae100_Supplemental_File [file nwae100_supplemental_file.pdf]

Supporting information for

Superparamagnetic iron oxide nanoparticle regulates microbiota-gut-inner ear axis for hearing protection

Zhanhang Guo[1,†], Yunhao Wu[2,3,†], Bo Chen[4], Mengdie Kong[3], Peng Xie[3], Yan Li[1], Dongfang liu[5,*], Renjie Chai[3,6,7,8,9,10,*], Ning Gu[1,11,*]

[1] Jiangsu Key Laboratory for Biomaterials and Devices, School of Biological Science and Medical Engineering, Southeast University, Nanjing 210009, China.

[2] Medical Science and Technology Innovation Center, Shandong First Medical University & Shandong Academy of Medical Sciences, Jinan 250000, China.

[3] State Key Laboratory of Digital Medical Engineering, Department of Otolaryngology Head and Neck Surgery, Zhongda Hospital, School of Life Sciences and Technology, School of Medicine, Advanced Institute for Life and Health, Jiangsu Province High-Tech Key Laboratory for Bio-Medical Research, Southeast University, Nanjing 210096, China.

[4] Institute of Materials Science and Devices, School of Materials Science and Engineering, Suzhou University of Science and Technology, Suzhou 215009, China.

[5] Jiangsu Key Laboratory of Molecular and Functional Imaging, Center of Interventional Radiology & Vascular Surgery, Department of Radiology, Medical School, Zhongda Hospital, Southeast University, No. 87, Dingjiaqiao, Nanjing 210009, China.

[6] Co-Innovation Center of Neuroregeneration, Nantong University, Nantong 226001, China.

[7] School of medical technology, Institute of Engineering Medicine, Beijing Institute of Technology, Beijing 100081, China.

[8] Department of Otolaryngology Head and Neck Surgery, Sichuan Provincial People's Hospital, University of Electronic Science and Technology of China, Chengdu, China.

[9] Institute for Stem Cell and Regeneration, Chinese Academy of Science, Beijing 100101, China.

[10] Southeast university Shenzhen research institute, Shenzhen 518063, China.
[11] Cardiovascular Disease Research Center, Nanjing Drum Tower Hospital, Affiliated Hospital of Medical School, Medical School, Nanjing University, Nanjing 210093, China.
These authors contributed equally: Zhanhang Guo, Yunhao Wu.
e-mail: Dongfang Liu: liudf@seu.edu.cn, Renjie Chai: renjiec@seu.edu.cn, Ning Gu: guning@nju.edu.cn.

METHODS

Materials

The materials and reagents such as sodium carboxymethyl cellulose (M.W. 90000), ferric chloride, ferrous chloride, ammonium aqueous solution (28%), sodium dihydrogen phosphate, potassium chloride, sodium chloride, glucose, glucuronic acid, urea, glucosamine hydrochloride, BSA, pepsin, mucin, NaOH solid and HCl aqueous solution (12 M) used in the experiments are all of chemically pure grade.

Synthesis of SPIOCA

A total of 300 mg of CMC was dissolved in 40 mL of ultrapure water, and 60 mg/30 mg, 150 mg/75 mg, or 300 mg/150 mg of hexahydrate ferric chloride and tetrahydrate ferrous chloride were dissolved in another 5 mL of ultrapure water. The two solutions were mixed in a three-necked flask and stirred under nitrogen flow (200 rpm) for 30 minutes. The water bath was heated to 60℃, and 5 mL of 14% ammonia solution was added using a microsyringe (1 mL/min) with vigorous stirring (800 rpm). The temperature of the water bath was then raised to 80℃ and maintained for 30 minutes before stopping the reaction. The resulting SPIOCA was obtained after workup by dialysis and filtration.

Characterization of SPIOCA

The morphology and size of iron oxide cores were observed using TEM (JEM-2100/FEI, Technai G20). The zeta potential was measured using a size and potential analyzer (Malvern, NanoZS90). X-ray diffraction (XRD) patterns of the powder in the range of 10°-80° 2θ were collected using an X-ray diffractometer (Smartlab3, Rigaku).

The hysteresis loop and saturation magnetization were obtained using a vibrating sample magnetometer (7407, lakesshore) at room temperature. FTIR spectra were obtained using an FTIR spectrometer (Nicolet IS10, Thermo Fisher). Elemental analysis was performed using transmission electron microscopy (Talos F200X, Thermo Fisher Scientific). The composition ratio of the nanoparticles was analyzed using a thermogravimetric analyzer (TG209F3, Tarsus). The iron content was detected using inductively coupled plasma mass spectrometry (Optima 5300DV, PE) and UV-visible spectrophotometry (UV-3600, Shimadzu, Japan). The pH value of the solution was measured using a pH meter (FE20, Metter Toledo).

Acid lability of SPIOCA

The compositions and concentrations of digestive fluids were referenced from the study by Agata P et al. (Supplementary Table 1) [1]. A total of 2 mL of SPIOCA (iron concentration of 1 mg/mL) or 2 mL of naked-Fe_2O_3 mixture was heated to 37°C and added to 28 mL of gastric fluid. The mixture was immediately transferred to a 37°C shaking incubator (100 rpm). At 30, 60, 120, 240, and 360 minutes, 5 mL of the mixture was removed and the dissolved iron was collected by centrifugation (6000 *g* for 10 minutes) and ultrafiltration (30K MWCO). The iron content in the filtrate was measured using the 1,10-phenanthroline colorimetric method and was calculated for the remaining particle proportion.

Animal experiments

C57BL/6 male mice were obtained from Gempharmatech Co., Ltd. (Nanjing, China). The experiments were performed after the mice were allowed to acclimate for 3 days. SPIOCA were administered intragastrically for 2 weeks, and then the mice were exposed to white noise (110 dB) for 2 hours in a soundproof chamber. The sound stimulus was generated by a Tucker Davies Technologies (TDT) system and amplified by high-fidelity speakers. Two weeks later, ABR tests and immunofluorescence analyses were carried out to detect the auditory function of mice. All animal experiments were approved by the Animal Care and Use Committee of Southeast University (approval number: 20210526003) and were consistent with the National

Institutes of Health Guide for the Care and Use of Laboratory Animals.

Toxicology of the SPIOCA *in vivo*

Twelve healthy C57BL/6 male mice were randomly divided into two groups, the control and SPIOCA groups. 200 μL Medium or SPIOCA (2.6 mg Fe/mL) were administered, respectively. In vivo biosafety observations were performed on mice for 14 days. At the indicated time, the mice were euthanized and their blood samples were collected for serum biochemical assays. The heart, liver, spleen, lung, kidney, stomach, jejunum, lleum, cecum and colon tissues of mice were collected and fixed with 4% paraformaldehyde (PFA) at 4°C for 24 hours. The PFA-fixed tissues were embedded in paraffin, sectioned continuously (5 μm), and stained with hematoxylin and eosin (H&E). The tissue morphology and pathology were observed under a microscope and analyzed.

ABR audiometry

ABR analysis was employed to measure the hearing thresholds of mice as described previously with minor modifications [2]. In brief, mice were anesthetized and placed on a pre-heated thermostatic pad (37 °C). The ABR waveforms were recorded at different frequencies (4, 8, 12, 16, 24 and 32 kHz) using subdermal needles that were placed over the vertex and bilaterally below the pinnae. The response signals were amplified and evaluated on a TDT System 3 apparatus (Tucker Davies Technologies, Gainesville, FL, United States).

Immunohistochemistry

After mice were sacrificed by intraperitoneal injection of excess pentobarbital sodium, the temporal bone was separated rapidly from the head in pre-cooled phosphate-buffered saline under a stereoscope. The samples were then fixed with 4% paraformaldehyde and decalcified with EDTA solution. The whole-mount preparations of the organ of Corti were dissected and adhered to a round glass slide that was coated with Cell-Tak (BD Biosciences). After washing three times with PBST for 5 min each time and blocking with 10% donkey serum for 2 h at room temperature, the samples were incubated with corresponding primary antibodies at 4°C overnight. The next day the samples were washed three times with PBST and incubated with the corresponding

secondary antibodies for 1 h at room temperature, then washed with PBST again and observed under a confocal microscope.

ELISA analysis

The levels of TNF-α, IL-17, and LPS in the colon and serum were measured using ELISA kits according to the corresponding manufacturer's instructions. The samples of each group were added to the ELISA plates after 5 times dilution. After co-incubation with conjugate reagent for 1 h at 37 °C, the supernatant was discarded and patted dry followed washing 5 times. Then the chromogenic agent was added to the ELISA plates for 15 min at 37 °C in dark. The levels of TNF-α, IL-17, and LPS were determined at 450 nm wavelength using a microplate reader (Tecan infinite M200 Pro, Switzerland).

Alcian blue staining

Colonic tissues were sectioned at 5 μm thickness, deparaffinized and stained using Alcian Blue staining kit (Leagene, Beijing, China) according to the manufacturer's instructions.

SPIOCA Distribution After Oral Administration

In summary, 5 ml of SPIOCA (3 mg/ml) was mixed with 0.5 ml of Cyanine 5.5 amine (dissolved in DMSO at 1 mg/ml). Subsequently, 5 mg of EDA and 5 mg of NHS were added, and the mixture was stirred in the dark for 2 hours. The product was purified by dialysis for 6 hours using a dialysis bag with a molecular weight cutoff of 3000, followed by ultrafiltration concentration, resulting in SPIOCA@cy5.5. SPIOCA@cy5.5 was orally administered to mice at the specified dose, and mice were euthanized and dissected at different time points (0h, 2h, and 6h). Organs including heart, liver, spleen, lungs, kidneys, stomach, small intestine, and colon were extracted, and the distribution of SPIOCA was observed using IVIS imaging.

ROS assessment

In this study, we employ 2',7'-dichlorodihydrofluorescein diacetate (DCFH-DA) as a fluorescent probe to detect reactive oxygen species (ROS) in colon tissue sections. 20 μM DCFH-DA was added to detect the intercellular ROS generation. After washing with PBS for three times, the tissue sections were qualitatively observed under confocal

microscopy and the ROS contents were quantitatively analysed by ImageJ software.

FITC-Dextran leakage assay

FITC-conjugated dextran was injected vail tail 1 h before sacrifice. Then the cochlea of mouse was isolated and processed into sections as described in the "Immunohistochemistry" section. The images were visualized with a confocal microscope. And dextran leakage was analyzed using the Image J software.

RNA sequencing

The mouse cochleae were collected and the total RNA was extracted using the FastPure Cell/Tissue Total RNA Isolation Kit V2 (Vazyme, RC112) according to the manufacturer's protocol. RNA integrity was evaluated using an Agilent 2100 Bioanalyzer (Agilent Technologies, Santa Clara, CA, USA). Libraries were constructed using a TruSeq Stranded mRNA LT Sample Prep Kit (Illumina, San Diego, CA, USA) according to the manufacturer's instructions. Sequencing of the libraries was performed on an Illumina X Ten platform and 150 bp paired-end reads were generated. Differential expression analysis was performed by DESeq R package [3]. GO enrichment and KEGG pathway enrichment analysis of differentially expressed genes were carried out using R based on the hypergeometric distribution.

16S rRNA gene amplicon sequencing

Bacterial DNA of colonic content samples was extracted using a DNA Extraction Kit (TIANGEN, DP328) following the manufacturer's instructions. The integrity and concentration of DNA were determined using a NanoDrop 2000 spectrophotometer. PCR amplification of the V3-V4 hypervariable regions of the bacterial 16S rRNA gene was conducted in a 25 μL reaction system with universal primer pairs (343F: 5'-TACGGRAGGCAGCAG-3'; 798R: 5'-AGGGTATCTAATCCT-3'). After purification with Agencourt AMPure XP beads (Beckman CoulterCo., USA) and quantification with Qubit dsDNA assay kit, the concentrations of the PCR products were adjusted for sequencing.

Non-targeted metabolomics

Samples were transferred to a 1.5 mL Eppendorf tube with L-2-chlorophenylalanine

dissolved in methanol as the internal standards. After storing at -20°C for 20 min and grinding for 2 min, samples were mixed with chloroform and vortexed. The whole mixtures were extracted by ultrasonication for 30 min in an ice-water bath, then placed at -20°C for 20 min and centrifuged at 4°C, 13000 rpm for 10 min. The supernatant was dried in a freeze concentration dryer, and the quality control sample was prepared by mixing aliquots of all the samples into a pooled sample. Methoxyamine hydrochloride in pyridine was added to the supernatant, and the resultant mixture was vortexed vigorously for 2 min and incubated at 37°C for 90 min. BSTFA (with 1% TMCS) and n-hexane were added into the mixture, the samples were then vortexed vigorously for 2 min and derivatized at 70°C for 1 h. The derivatized samples were analyzed on an Agilent 7890B gas chromatography system coupled to an Agilent 5977A MSD system (Agilent Technologies Inc., CA, USA).

Statistical analysis

All of the obtained values are expressed as mean ± standard deviation (SD) from at least three independent experiments. All data are analyzed with GraphPad Prism9. The statistical significance was determined with Student's t-test for comparisons between two groups or one-way analysis of variance (ANOVA) for multiple comparisons and $p < 0.05$ was considered statistically significant.

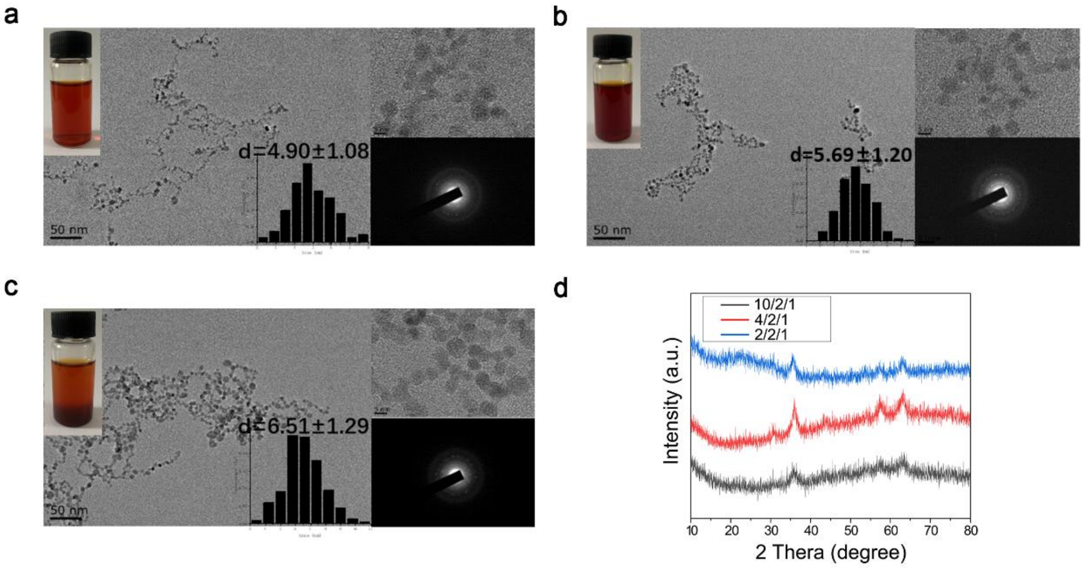

Figure S1. TEM images, size distributions, and SAED patterns of SPIOCA prepared at different precursor ratios. The molar ratio of CMC/ $FeCl_3 \cdot 6H_2O$ / $FeCl_2 \cdot 4H_2O$ is (a) 10/2/1, (b) 4/2/1, and (c)2/2/1, (d) XRD pattern of three samples.

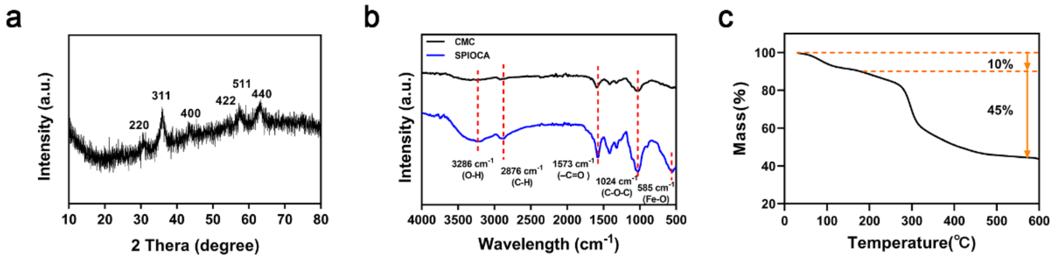

Figure S2. Structure characterization of SPIOCA. (a) XRD pattern of SPIOCA. (b) FTIR absorption spectra of CMC and SPIOCA. (c) Thermogravimetric analysis of SPIOCA.

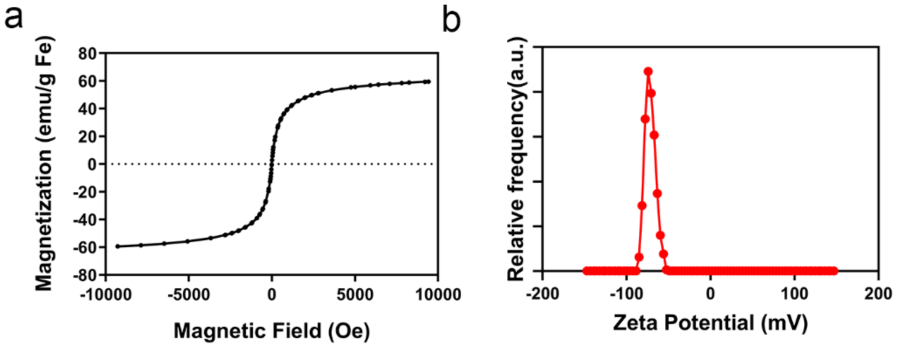

Figure S3. Physical properties of SPIOCA. (a) Field-dependent magnetization curves of SPIOCA. (b) Zeta Potential of SPIOCA.

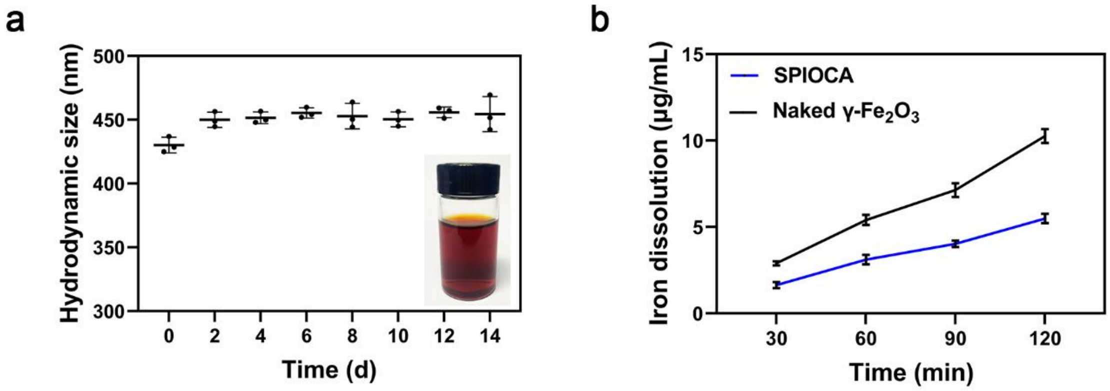

Figure S4. Stability of SPIOCA. (a) Hydrodynamic diameter of SPIOCA over 14 days. (b) Iron ion release of SPIOCA and naked γ-Fe_2O_3 in simulated gastric juice.

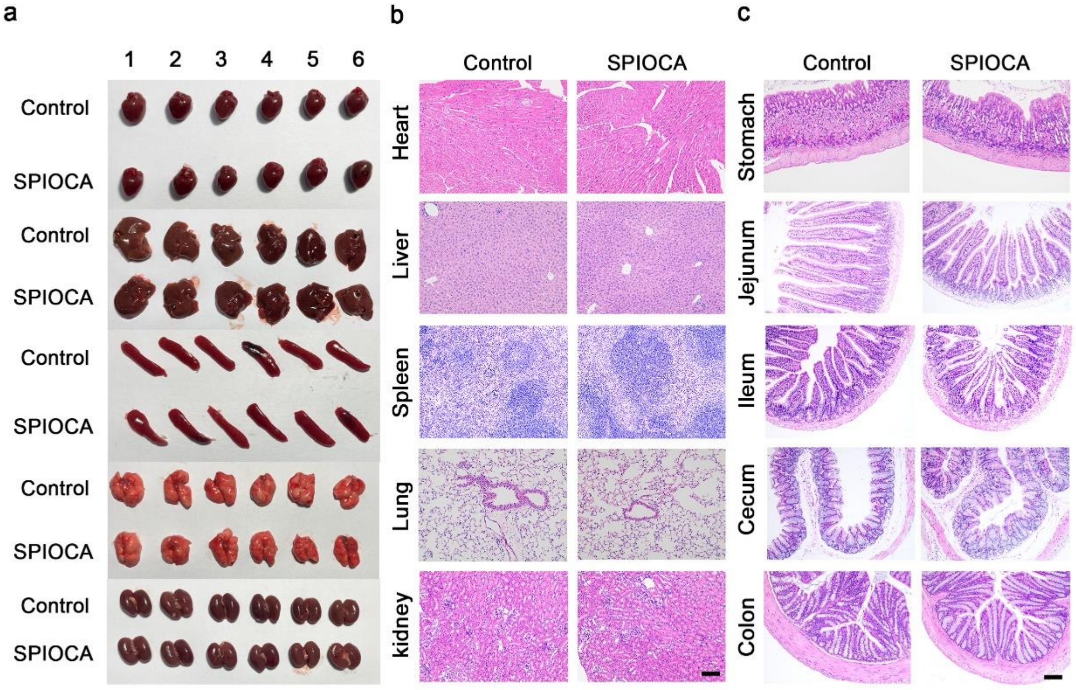

Figure S5. Biosafety evaluation of SPIOCA through H&E analysis. (a) Representative photographs and (b) H&E images obtained from the major organs of healthy mice orally administered with or without SPIOCA for 14 days ($n = 6$). Scale bars, 100 μm.

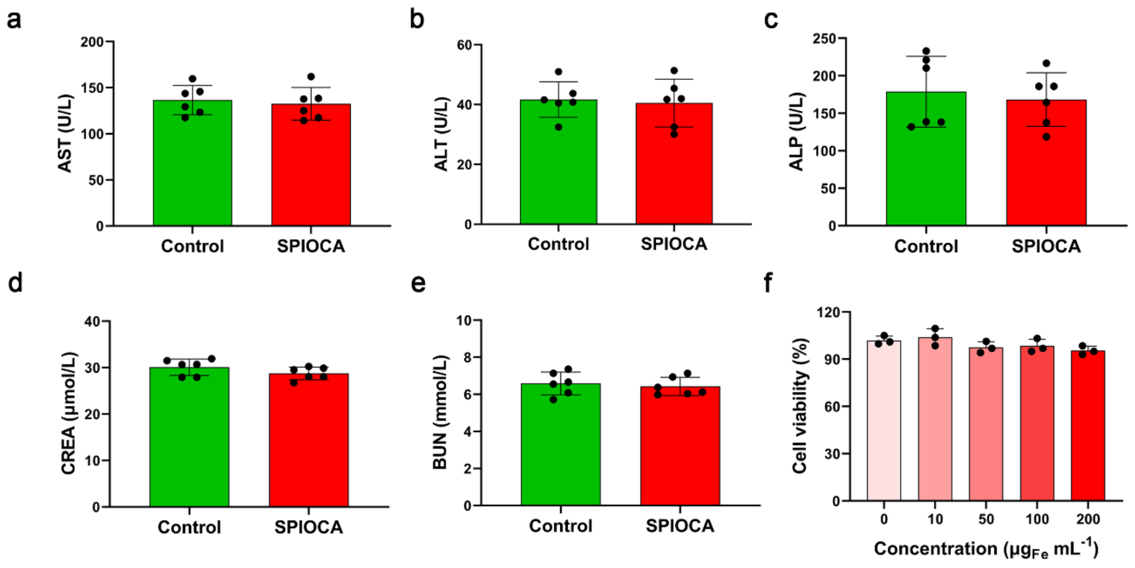

Figure S6. Biosafety evaluation of SPIOCA. (a-e) Hematological analysis (AST, ALT, ALP, CREA, BUN) of healthy mice orally administered with or without SPIOCA for 14 days. (f) Cell viability of Caco-2 cells treated with different concentrations of SPIOCA for 24 h.

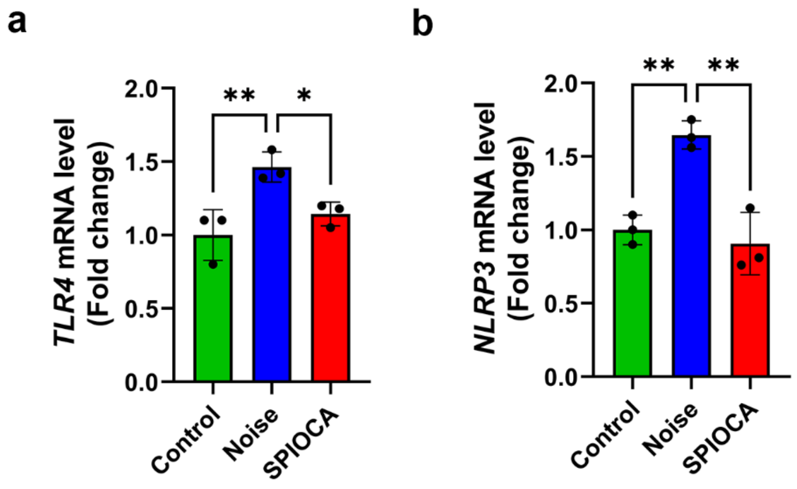

Figure S7. Determination of the mRNA levels of *TLR4* and *NLRP3* in cochlea. (a) The *TLR4* mRNA level in different groups (n = 3). (b) The *NLRP3* mRNA level in different groups (n = 3). *$P < 0.05$, **$P < 0.01$.

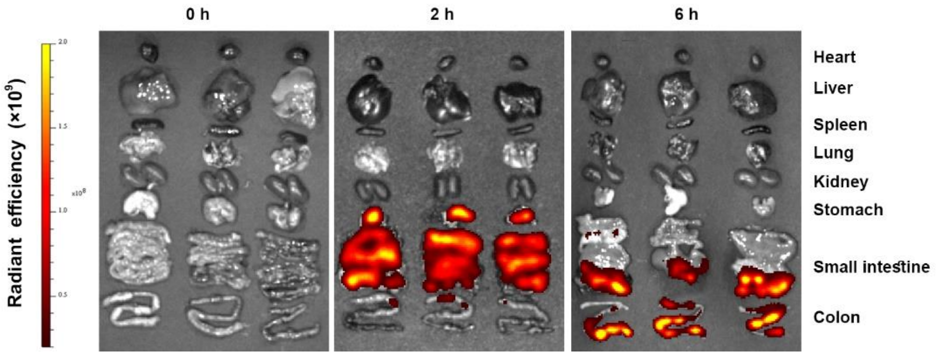

Figure S8. IVIS imaging of organs at different time points following oral administration of SPIOCA@cy5.5.

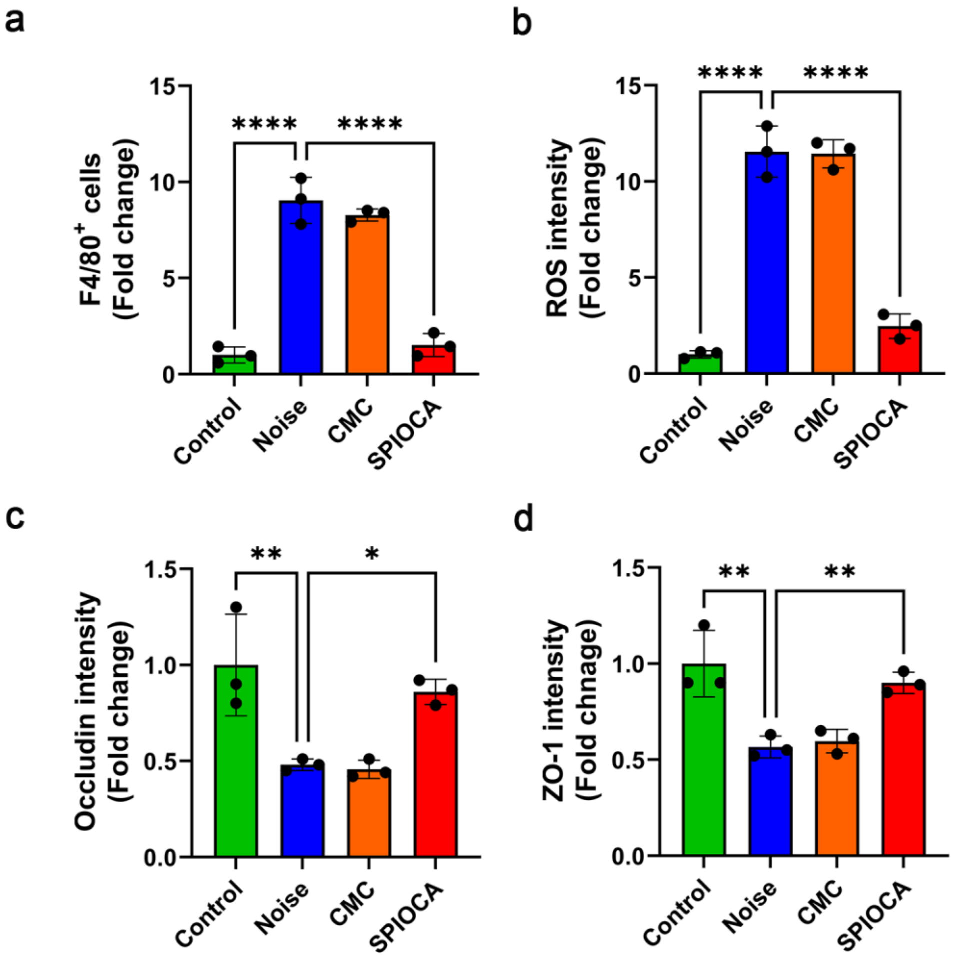

Figure S9. Quantification of F4/80$^+$ cells (a), ROS level (b), occluding (c) and ZO-1 (d) expression levels in Fig. 5. *$P < 0.05$, **$P < 0.01$, ****$P < 0.0001$.

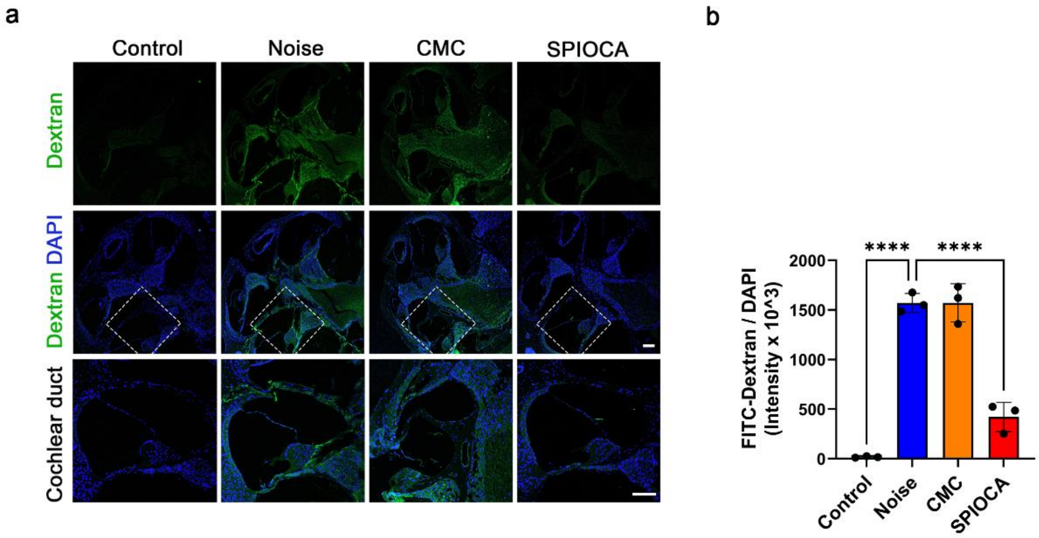

Figure S10. SPIOCA protects against noise-induced blood-labyrinth barrier disruption. (a) Representative immunofluorescence image of cochlea sections of different groups injected with FITC-labeled dextran. DAPI represents nuclear signal (blue). Scale bars, 100 μm. (b) Quantification of dextran-containing cells per DAPI in the cochlea. ****$P < 0.0001$.

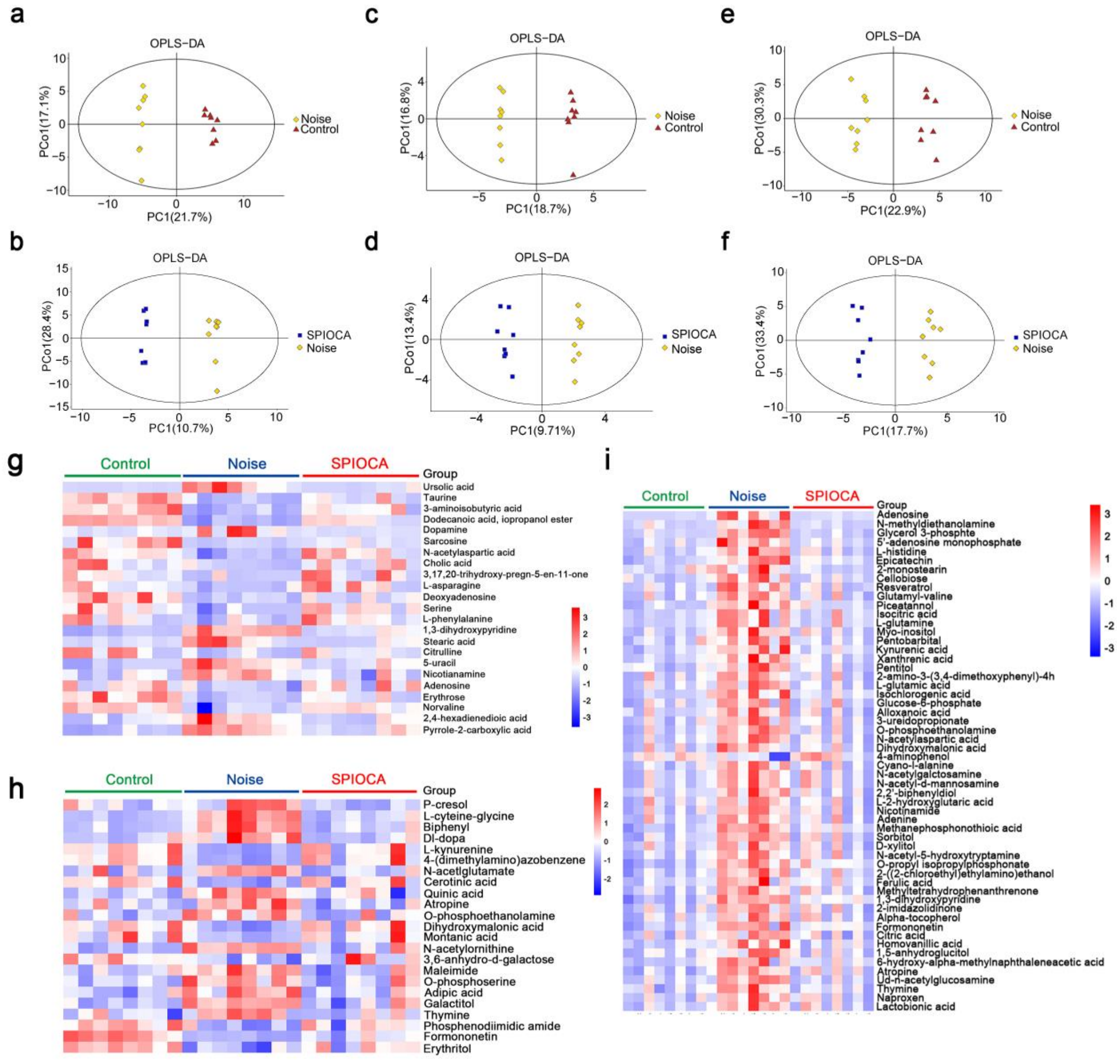

Figure S11. SPIOCA alters metabolic profiling in the colon, serum and brain. (a and b) Orthogonal partial least squares-discriminant analysis (OPLS-DA) of metabolomic profiles in colonic contents (n = 8) of the Control vs. Noise and Noise vs. SPIOCA groups. (c and d) OPLS-DA of metabolomic profiles in serum (n = 8) of the Control vs. Noise and Noise vs. SPIOCA groups. (e and f) OPLS-DA of metabolomic profiles in brain (n = 8) of the Control vs. Noise and Noise vs. SPIOCCA groups. (g-i) Heatmap of significantly altered metabolites in colonic contents, serum, and brain (n = 8).

Supplementary Table 1. Composition of gastric Juices (Amounts Based on 1000

mL of Juice).

pH	Inorganic constituents	Organic constituents
	2752 mg NaCl	85 mg urea
	306 mg $NaH_2PO_4 \cdot H_2O$	330 mg glucosaminhydrochloride
	824 mg KCl	1 g BSA
1.3±0.1	302 mg $CaCl_2$	2.5 g pepsin
	6.5 mL 37% HCl	3 g mucin
	650 mg glucose	Milli-Q water
	20 mg glucuronic acid	

REFERENCES

1. Walczak AP, Fokkink R and Peters R *et al*. Behaviour of silver nanoparticles and silver ions in an human gastrointestinal digestion model. *Nanotoxicology* 2013; **7**: 1198-210.
2. Liu Y, Qi JY and Chen X *et al*. Critical role of spectrin in hearing development and deafness. *Sci Adv* 2019; **5**: eaav7803.
3. Anders S and Huber W. Differential expression of RNA-Seq data at the gene level– the DESeq package. *EMBL* 2012; **10**: f1000research.
